# Hiatal hernia repair: prevention of mesh erosion and migration into the esophagogastric junction

**DOI:** 10.1590/0102-672020190001e1489

**Published:** 2020-05-18

**Authors:** Italo BRAGHETTO, Owen KORN, Jorge ROJAS, Hector VALLADARES, Manuel FIGUEROA

**Affiliations:** 1 Department of Surgery, Hospital Dr. José J. Aguirre, Faculty of Medicine, University of Chile, Santiago, Chile.

**Keywords:** Hiatal hernia, repair, Mesh erosion, prevention, Hénia hiatal, Laparoscopia, Telas cirúrgicas, Prevenção

## Abstract

***Background*:**

Erosion and migration into the esophagogastric lumen after laparoscopic hiatal hernia repair with mesh placement has been published.

***Aim*:**

To present surgical maneuvers that seek to diminish the risk of this complication.

***Method*:**

We suggest mobilizing the hernia sac from the mediastinum and taking it down to the abdominal position with its blood supply intact in order to rotate it behind and around the abdominal esophagus. The purpose is to cover the on-lay mesh placed in “U” fashion to reinforce the crus suture.

***Results*:**

We have performed laparoscopic hiatal hernia repair in 173 patients (total group). Early postoperative complications were observed in 35 patients (27.1%) and one patient died (0.7%) due to a massive lung thromboembolism. One hundred twenty-nine patients were followed-up for a mean of 41+28months. Mesh placement was performed in 79 of these patients. The remnant sac was rotated behind the esophagus in order to cover the mesh surface. In this group, late complications were observed in five patients (2.9%). We have not observed mesh erosion or migration to the esophagogastric lumen.

***Conclusion*:**

The proposed technique should be useful for preventing erosion and migration into the esophagus.

## Graphical Abstract

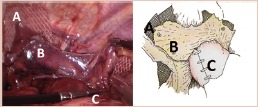

## RESUMO

***Racional*:**

Com a colocação de tela foi têm sido publicadas erosões e migrações para o lúmen esofagogástrico após correção de hérnia hiatal laparoscópica.

***Objetivo*:**

Apresentar manobras cirúrgicas que buscam diminuir o risco dessa complicação.

***Método*:**

Sugerimos mobilizar o saco de hérnia do mediastino e levá-lo à posição abdominal com o suprimento sanguíneo intacto, a fim de girá-lo para trás e ao redor do esôfago abdominal. O objetivo é cobrir a malha colocada sobre a forma “U” para reforçar a sutura da crura haital.

***Resultados*:**

Realizamos reparo laparoscópico de hérnia hiatal em 173 pacientes (grupo total). Complicações pós-operatórias precoces foram observadas em 35 pacientes (27,1%) e um morreu (0,7%) devido a tromboembolismo pulmonar maciço. Cento e vinte e nove pacientes foram acompanhados por média de 41+28 meses. A colocação da tela foi realizada em 79 desses pacientes. O saco remanescente foi girado atrás do esôfago para cobrir a superfície da tela. Nesse grupo, complicações tardias foram observadas em cinco pacientes (2,9%). Não observamos erosão da tela ou migração dela para o lúmen esofagogástrico.

***Conclusão*:**

A técnica proposta pode ser útil para prevenir a erosão e a migração para o esôfago de telas na correção de hérnias hiatais.

## Graphical Abstract

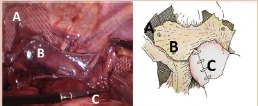

## INTRODUCTION

A high recurrence rate after laparoscopic hiatal hernia repair, which can reach up to 66%, ranging from 1.2% to 66%^1^ ^,^ ^12^ ^,^ ^16^ ^,^ ^17^ ^,^ ^19^ ^,^ ^27^, has been reported in patients with giant type III or IV hernias. In order to diminish this recurrence after surgery, different types of mesh have been proposed^5^. Polypropylene, polyester, polytetrafluoroethylene (PTFE), biological mesh, and different types of dual mesh are the most common types that have been used. In addition, a vast variation in mesh configuration and positioning has also been employed^11^. Some of these products carry a risk of migration into the esophagogastric lumen. Biomaterial tends to be associated with failure and a high rate of recurrence, but it does not present risk of migration, whereas non-absorbable mesh tends to be associated with stricture and erosion. Erosion and esophageal stricture due to dense fibrosis, (range from 0.3% to 2%), have been reported. Dual mesh or other composed mesh have been used in order to avoid this complication^6^ ^,^ ^11^. 

In this article, we present our technique to prevent or diminish the risk of erosion of the esophagogastric wall and migration into the lumen when non-absorbable mesh is used.

## METHOD

The authors declare that no experiments were performed on humans or animals for this study. Confidentiality data have followed the protocols of their work center on the publication of patient data and, based on right to privacy and informed consent, the authors declare that no patient data appears in this article. 

### Patients

From January 2007 to December 2016, our department operated on 961 patients diagnosed with gastroesophageal reflux and hiatal hernia. One hundred seventy-three of them corresponded to a giant hiatal hernia, with a mean age of 69.5 years (34-84), and they were subjected to hiatal hernia repair. Giant type III or IV hiatal hernias were defined as hiatal hernias larger than 10 cm in size. These were diagnosed by measuring the axial and transverse diameters during the radiologic examination (barium swallow) and subsequently confirmed during the laparoscopic exploration^9^. In Table 1 the characteristics of these patients are shown. Only one patient presented an index of obesity and four ASA III category due to medical co-morbidities (arterial hypertension, chronic asthma, over 70 years of age, type II diabetes). In 79 of these patients, mesh placement was performed and the remnant sac was rotated behind the esophagus in order to cover the mesh surface. 

**TABLE 1:** Demographic characteristics of patients submitted to laparoscopic hiatal hernia repair (n=173)

Age:	mean	69.5 years (range:34-84 years)
Gender:	Female	136 (75.9%)
	Male	43 (24.1%)
Weight:	mean:	71.3 Kg (range 59- 91kg)
Body mass index	(BMI) mean:	29.8Kg/m2
Obese patient:	1 (BMI 36.4 with arterial hypertension, dyslipidemia and bilateral safeneus varices)	
ASA score:	ASA I	141 (78.8%)
	ASA II	36 (20.1%)
	ASA III	2 (1.1%)
Hernia type:	I	101 (56.4%)
	II	6 (3.3%)
	III	51 (28.5%)
	IV	21 (11.7%)
Hernia size (cm):	10 -15	99 (55.3%)
	16-20	59 (32.9%)
	> 21	21 (11.7%)

### Surgical technique

After 15 mmHg of intra-abdominal pneumoperitoneum, five trocars were inserted: the first, 10 mm in diameter, at midline 3 cm above the umbilicus for the optical system; one 5 mm in sub-xiphoid point for the liver retraction; one 5 mm in the right subcostal medium clavicular line; one 10 mm trocar in the left anterior axillary line and another 10 mm trocar in the left medium clavicular line for working ports.

The proper technique that was employed is described step by step, with additional figures to clarify the maneuvers demonstrating the procedure. This consisted of the following steps:

#### Dissection of hernia sac

**FIGURE 1:**
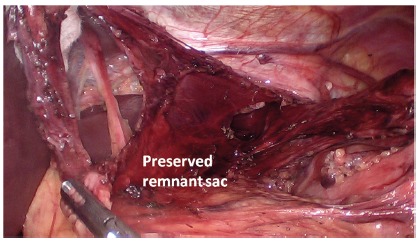
Preservation of hiatal hernia sac: once the sac was completely dissected from the mediastinum, it was brought down from the mediastinum and its blood supply was kept attached to the lesser gastric omentum.

Began 2 cm behind the left crus on the mediastinal reflection, leaving a small portion of the sac adhered to the crus in order to avoid exposure of uncovered muscle fibers. The dissection continued towards the right crus exposing the anterior face of the esophagus, identifying the anterior trunk of the vagus nerve (which must be preserved) in order to obtain complete mobilization of the sac in the lateral, anterior and posterior area of the distal esophagus (Figure 1). Once the lateral and posterior face of the distal esophagus and esophagogastric junction were isolated and could be easily mobilized, they were placed in the abdominal cavity at least 2 cm below the hiatus, completely free of tension (we have never observed a short esophagus). The first short gastric vessels were also divided and in this manner, both the left and right diaphragmatic crus were clearly exposed. A window through the avascular membrane of the lesser gastric omentum (gastro-hepatic omentum) and another small window above the hepatic branches of the anterior vagus nerve (which remain intact) were performed thus completing the visualization of the dissected right crus.

#### Closure of the hiatus

Closure of the diaphragmatic crus with a posterior approach behind the esophagus was performed, using 2 to 3 non-absorbable interrupted sutures. Frequently, anterior closure of the pillars may also be required with additional stitches depending on the hiatus’s diameter in order to avoid angulation of the distal esophagus at the hiatal passage. In giant hiatal hernias, an on-lay “U” shaped mesh is placed over the posterior closure of the crura using non-absorbable 5 cm mesh (Parietex^®^ or Ultrapro^®^). In order to maintain both branches of the mesh separated, the mesh must be fixed with either intracorporeal sutures or tackers (depending on its availability) over the muscle area of both cruses (not over the diaphragm itself in order to avoid pericardial or cardiac injury).

#### Management of hernia sac

**FIGURE 2:**
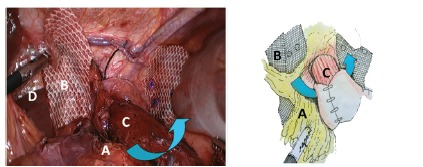
The arrows show how the preserved hernia sac was rotated and passed behind the esophagogastric junction and over the crura closure in order to cover the mesh. The right branch of mesh was placed lateral to the dissected right crus, behind the hepatic branches of the anterior vagus nerve, which were preserved. In this manner, both branches of the mesh remain separated and covered.

In order to cover the mesh, prevent or minimize the risk of esophageal or gastric wall erosion and migration of mesh: a) during dissection, the preserved remnant sac was placed behind the esophagogastric junction. We preserved almost the entire dimension of this sac (at least 5x3 cm, mean area of 15 cm^2^) which remained with its vascular supply from the lesser omentum vessels intact; b) this remnant sac was then rotated around to the esophagogastric junction and fixed with sutures that widely covered the mesh surface (Figure 2); c) in this manner, the mesh was covered in order to prevent late migration or erosion of the mesh into the esophagus. 

A=remnant sac rotation passing behind the esophagus; B=mesh; C=esophagus; D=right crus dissection above the preserved hepatic branches of the anterior vagus nerve.

#### Fundoplication

A calibrated Nissen fundoplication over a 36F bougie was performed with non-absorbable sutures and the distal esophagus was wrapped. A posterior gastropexy of the wrap to the sutured crus was performed in addition to an anterior fundo-phrenopexy to prevent its anterior migration. 

### Follow-up

One hundred twenty-nine patients (82.3%) completed a mean follow-up of 41+28 months, with the maximal follow-up being 12 years, so that clinical recurrence could be evaluated, and 79 of these corresponded to patients in whom the described technique was applied. 

Patients were controlled periodically at six months and 1, 2, 3 or more years after surgery and the mean follow-up of patients included in this study was 41+28 months (18-144). The presence of postoperative reflux symptoms (heartburn, regurgitation) and dysphagia were investigated using a standardized questionnaire. If reflux symptoms were detected, the endoscopy and barium sulphate swallow were repeated after surgery in order to confirm the objective recurrence of hiatal hernia. The clinical control of symptoms was conducted by the first author (IB), and endoscopic and radiological examinations were performed. The results of these studies were recorded in the Tycares^®^ database system of our institution. 

**FIGURE 3:**
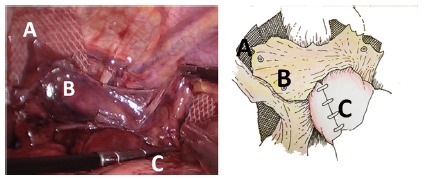
Both fixed branches of mesh were completely covered with the rotated preserved sac: A) fixed mesh; B) preserved sac covering the mesh; C) fundoplication

## RESULTS

During the period of study, 173 patients were submitted to laparoscopic hiatal hernia repair due to giant hiatal hernia with a mean operative time of 183 min (160-205). Postoperative complications are shown in Table 2. Major early postoperative complications, mainly respiratory (pleural effusion, atelectasis), were observed in 18 patients (10.1%). Six were early reoperated, being four due to hemoperitoneum (one spleen injury), and two due to esophageal perforation. One patient with concomitant obesity died (0.6%) due to a massive lung thromboembolism on the 3^rd^ postoperative day. 

**TABLE 2:** Early postoperative complications observed in patients with giant hiatal hernia submitted to hiatoplasty plus mesh placement (n=179)

	N	%	Management
Respiratory complications	7	4.1	Medical treatment
Hemoperitoneum	4	1.7	Reoperation
Esophageal perforation	2	1.2	Reoperation
Mediastinal collection	2	1.2	1 reoperation, 1 medical treatment
Lung thromboembolism	2	1.2	Medical treatment
Gastric retention	1	0.8	Medical treatment
Total	18	10.1	
Mortality	1	0.6	(3rd postoperative day due to lung thromboembolism)

Table 3 shows the late complications observed in 79 patients submitted to the proposed procedure. The most common, dysphagia, was observed in 12 patients (15.2%). They were initially managed with endoscopic dilatation. Applying our proposed score^1^, based on the presence of symptoms and presence of hiatal hernia >5 cm size determined by radiological or endoscopic evaluation, a “true” recurrence was observed in 33 patients (41.8%). If a patient was asymptomatic and with a hernia <5 cm, relative recurrence or no recurrence was considered. Late reoperation was needed in seven patients (5.4%), three of them due to persistent dysphagia (3.8%). The others remained under medical treatment with proton pump inhibitors. Among these 79 patients submitted to technique proposed, until now we have not observed erosion or migration of mesh into the esophagogastric lumen, even after 12 years follow-up.

**TABLE 3:** Late complications observed during the follow-up in patients with giant hiatal hernia submitted to hiatoplasty plus mesh placement and rotation of remnant hernia sac in order to cover the mesh (n=79)

	N	%	Treatment
Dysphagia	1	15.2	Endoscopic dilatation
Stricture	3	3.8	Reoperation
Recurrence			
Radiological/endoscopic	33	41.8	
Symptomatic recurrence with esophagitis	10	12.7	
Type A	6		
Type B	1		
Type C	3		
			7 reoperated (5.4%)
			3 due to strictures
Persistent Diarrhea	2	2.5	Medical treatment
Late erosion or migration of mesh	0		

## DISCUSSION

The technique for laparoscopic hiatal hernia repair has been well established. However, it merits some comments because some surgeons do not completely dissect the hernia sac and leave part of the sac in situ. On the contrary, others perform complete resection because un-resection of sac could be a factor for hernia recurrence^18^. We propose to dissect the sac, perform its mobilization from the mediastinum and bring it down to the abdominal position maintaining its blood supply intact. The purpose is to rotate it behind and around the abdominal esophagus so as to cover the on-lay mesh placed in “U” fashion, similarly to a laparoscopic inguinal hernia repair with a trans-peritoneal approach. Our procedure is entirely different from the technique without dissection of sac from the mediastinum, because we perform complete mobilization of the hernia sac from the mediastinal space to the abdomen; however, it remains adhered and vascularized below the diaphragmatic crura. Therefore, although it is unlikely to find retraction or slippage in the mediastinum, we cannot exclude the possibility of increasing the rate of recurrence in long term (>10 years) follow-up. We completely avoid any overlapping of the mesh, because it´s retraction in contact with the esophageal wall could favor the appearance of stricture, erosion and its migration into the esophagogastric lumen. True short esophagus is rare. We have not observed short esophagus in our experience because after complete esophageal dissection and isolation, it is possible to obtain an abdominal esophageal segment more than 2 cm free of tension.

In the available literature, our proposed maneuvers have not always been reported. On the contrary, most surgeons even completely divide the gastro-hepatic omentum and hepatic branches of the anterior vagus nerve. However there is late risk of gallstones. On the contrary we preserve the hepatic vagal branches and we perform only a window on the gastrohepatic ligament as was describe. This maneuver is useful because, first, preservation of vagus nerve trunks is obtained thus avoiding its damage and the subsequent delayed gastric emptying and development of gallstones after surgery; secondly, it allows to adequately place the mesh and cover it with the sac remnant (most surgeons do not pay attention to this); and third, fixation of the right branch of the “U” shaped mesh can also be performed easily and far from the esophageal wall. 

Short-term symptomatic results are excellent, but mid-term or long-term follow up objective results, observed in patients who have been submitted to a hernioplasty with and without mesh placement, demonstrate a high rate of recurrence ranging from 10-66%.

However, recurrence is less frequent after a hiatal hernioplasty with mesh. In a recent publication, hernia recurrence was reported in 23.1% after suture repair, 30.8% after absorbable mesh, and 12.8% after non-absorbable mesh^25^. It is very important to consider the size of the herniated stomach and the hernia surface area, as suggested by Granderath et al.^7^, because hiatal hernias larger than 10 cm in diameter have a higher rate of recurrence^2^ ^,^ ^6^ ^,^ ^7^ ^,^ ^15^. The Nebraska group presented a follow-up of 209 patients in which they demonstrated high recurrence rates that increased over time from 16% at one year, up to 40% after five years^13^. This high recurrence could be multi-factorial, due to patients´ basal conditions, surgical technique, type of mesh placement, follow-up and also due to its variation in the endoscopic, radiological and symptomatic definition. Some authors have suggested that despite frequent radiologic recurrence, symptoms are well tolerated and patient satisfaction is very high. Preoperative symptoms improved in 70% of patients and reoperations were very low^16^ ^,^ ^17^. Regarding the definition of hernia recurrence, our current opinion is that both images and the associated symptoms must be taken into account. Our recurrence rate is comparatively less than that of the literature because we consider “true” recurrence only if a patient is symptomatic and with a recurrent hernia >5 cm size. According to our published score, if a patient is asymptomatic with a hernia <5 cm, a relative recurrence or no recurrence is considered^1^. This is the reason why our recurrence appears to be less than in other publications. 

Mesh placement can be associated with severe complications secondary to erosion and migration of mesh into the lumen, such as esophageal ulcer and stricture due to dense fibrotic tissue. The appearance of these complications is mainly very late (after five years) but there are cases in which migration occurred before three years after the operation^8^ ^,921,^ ^22^ ^,^ ^24^ ^,^ ^26^. For some authors, esophageal erosion occurred in few cases (0.49%)^4^ ^,^ ^9^ ^,^ ^14^ ^,^ ^23^ ^,^ ^28^, but is difficult to establish the exact rate of erosion, migration or stricture because these complications are not always reported. In a survey conducted on 165 European surgeons, esophageal erosions were encountered by 33 (20.0%) and esophageal stenosis due to dense fibrosis by 34 of them (20.6%)^11^ ^,^ ^15^. The main symptoms were dysphagia, heartburn, chest pain, fever, epigastric pain, weight loss, and some patients required an esophagectomy, a partial gastrectomy and even a total gastrectomy as treatment^23^. Therefore, is important to avoid this discouraging clinical situation. Mesh fixation distant from the esophagogastric wall may be important to avoid late esophageal wall injury.

The limitation of this study is that there is no follow-up greater than 10 years for the complete group of included patients. 

## CONCLUSION

We believe that the proposed procedure accomplishes the purpose of preventing mesh erosion and migration into the esophagogastric junction. Up to now, after late follow-up, we have not observed them in our patients. 
